# Long non-coding RNA DLGAP1 antisense RNA 1 accelerates glioma progression via the microRNA-628-5p/DEAD-box helicase 59 pathway

**DOI:** 10.1016/j.clinsp.2021.100002

**Published:** 2022-02-02

**Authors:** Ke-qi Hu, Xiang-sheng Ao

**Affiliations:** Department of Neurosurgery, Xiangyang Central Hospital, Affiliated Hospital of Hubei University of Arts and Science, China

**Keywords:** Glioma, *DLGAP1-AS1*, *miR-628-5p*, *DDX59*

## Abstract

**Objectives:**

Abnormal expression of long non-coding RNAs (lncRNAs) plays a prominent role in glioma progression. However, the biological function and mechanism of lncRNA DLGAP1 antisense RNA 1 (*DLGAP1-AS1*) in gliomas are still unknown.

**Methods:**

The authors assessed *DLGAP1-AS1* and *miR-628-5p* expression in glioma tissues and cell lines using quantitative real-time polymerase chain reaction (qRT-PCR) and evaluated their effects on glioma cell proliferation, migration, invasion, and epithelial-mesenchymal transition (EMT) using the cell counting kit-8 (CCK-8) assay, 5-Ethynyl-2′-deoxyuridine (EdU) assay, Transwell assay, and western blot, respectively. The expression of DEAD-box helicase 59 (*DDX59*) was quantified using western blotting, and a dual-luciferase reporter gene assay was performed to detect the interaction between *DLGAP1-AS1* and *miR-628-5p*.

**Results:**

The authors observed increased *DLGAP1-AS1* expression in glioma tissues and cell lines with higher WHO grades and shorter survival time. *DLGAP1-AS1* promoted the proliferation, migration, invasion, and EMT of glioma cells, while *miR-628-5p* counteracted these effects. The authors identified *DLGAP1-AS1* as a molecular sponge of *miR-628-5p* in glioma cells as the biological functions of *DLGAP1-AS1* are partially mediated via *miR-628-5p*. In addition, *DLGAP1-AS1* upregulated *DDX59* expression by inhibiting *miR-628-5p* expression.

**Conclusion:**

The *DLGAP1-AS1*/*miR-628-5p*/*DDX59* axis regulates glioma progression.

## Highlights

• The expression of DLGAP1-AS1 expression is increased in glioma.
• DLGAP1-AS1 promotes the malignancy of glioma via modulating miR-628-5p/DDX59 axis.

## Introduction

Glioma – a cancer of the brain and spinal cord – has a high recurrence rate, morbidity and mortality, and poor prognosis.^1^, ^2^, ^3^ Elucidating the mechanism of glioma progression has great significance with respect to improving the prognosis of patients with this deadly disease.

Long non-coding RNAs (lncRNAs) are ≥ 200 nt in length, lack protein-coding capabilities but are involved in regulating biological processes, such as gene imprinting, RNA splicing, and chromatin modification.^4^, ^5^, ^6^ LncRNAs can control gene expression at the transcriptional and post-transcriptional levels and thus play key roles in cancer biology.^7^ In addition, as lncRNA expression exhibits tissue specificity,^8^ they may serve as biomarkers and potential therapeutic targets. For example, lncRNA *ATB* promotes the growth of gastric cancer by regulating the *miR-141-3p*/TGF-β2 axis.^9^ LncRNA *SIK1-LNC* inhibits the proliferation and metastasis of lung cancer cells, and its expression is downregulated in lung cancer.^10^ Recently, lncRNA DLGAP1 antisense RNA 1 *(DLGAP1-AS1)* has been found to act as an oncogenic lncRNA with its expression upregulated in hepatocellular carcinoma.^11^^,^^12^ However, little is known about the role of *DLGAP1-AS1* in gliomas.

MicroRNAs (miRNAs) – small non-coding RNAs approximately 21–25 nt in length – are crucial players in cancer biology.^13^, ^14^, ^15^, ^16^ They can repress gene expression by binding to the 3ʹ-untranslated region (3’-UTR) of an mRNA, regulating various physiological and pathological processes.^17^ Reportedly, *miR-628-5p* represses the malignant phenotypes of glioma cells by targeting high mobility group protein B3 (*HMGB3*) and DEAD-box helicase 59 (*DDX59).* It is downregulated in glioma tissues and cells, indicating that *miR-628-5p* is a tumor suppressor in the brain.^18^^,^^19^

In the present study, the authors investigated the expression patterns, biological functions, and mechanisms of action of *DLGAP1-AS1* in gliomas. The authors found that *DLGAP1-AS1* expression was significantly upregulated in glioma tissues and cell lines, and it promoted the proliferation, migration, invasion, and Epithelial-Mesenchymal Transition (EMT) of glioma cells. Mechanistically, *DLGAP1-AS1* functions as a competitive endogenous RNA (ceRNA) to sponge *miR-628-5p* and upregulate DDX59 expression. The present study proposes a novel ceRNA network for glioma progression.

## Material and methods

### Tissues collection

Tissue samples were obtained from patients who underwent surgery in the Department of Neurosurgery, Xiangyang Central Hospital, from May 2014 to July 2018, including 59 glioma tissue samples and 59 corresponding adjacent non-tumor tissue samples. After removal, the samples were stored in liquid nitrogen at -196°C. This study followed the 2007 World Health Organization (WHO) classification of tumors of the central nervous system (WHO grade I, n = 12: pilocytic astrocytomas (n = 8) and myxopapillary ependymomas (n = 4); grade II, n = 23: diffuse astrocytomas (n = 17), oligoastrocytomas (n = 3), and oligodendrogliomas (n = 3); grade III, n=14, anaplastic astrocytomas (n=6), anaplastic oligodendrogliomas (n = 5), and anaplastic oligoastrocytomas (n = 3); grade IV, n = 10: glioblastomas). The tumor samples were divided into low-grade tumors (grade I and II, n = 35) and high-grade tumors (grade III and IV, n = 24).^20^ This study was approved by the Research Ethics Committee of the Xiangyang Central Hospital. Written informed consent was obtained from each patient.

### Cell lines and cell culture

The Cell Bank of Shanghai Institutes for Biological Sciences, Chinese Academy of Sciences (Shanghai, China) provided the glioma cell lines (U-118MG, U251, U87MG, and LN229 cells), astroglia cell line (HA cells), and human embryonic kidney cell line (HEK-293 cells). The cells were maintained in Dulbecco's modified Eagle's medium (DMEM, Thermo Fisher Scientific, Waltham, MA, USA) supplemented with 10% fetal bovine serum (FBS, Gibco, Carlsbad, CA, USA), 100 U/mL penicillin, and 100 μg/mL streptomycin (Gibco, Carlsbad, CA, USA) in 5% CO_2_ at 37°C.

### Quantitative real-time polymerase chain reaction (qRT-PCR)

To determine *DLGAP1-AS1* and *DDX59* expression, the authors extracted the total RNA from tissues or cells using TRIzol reagent (Invitrogen, Carlsbad, CA, USA), reverse transcribed cDNA using a reverse transcriptase kit (Takara, Dalian, China), and performed qRT-PCR with SYBR Green Master Mix (Takara, Dalian, China). The NormFinder program was used to determine the appropriate housekeeping genes for the normalization of qRT-PCR data. After evaluating *GAPDH* (M-value = 0.518) and *ACTB* (M-value=0.646), the authors used them as the endogenous controls.^21^ To determine *miR-628-5p* expression, qRT-PCR was performed using a TaqMan miRNA reverse transcription kit (Applied Biosystems, Grand Island, NY) with U6 and U48 as endogenous controls. Relative expression levels of *DLGAP1-AS1, DDX59,* and *miR-628-5p* were estimated using the 2^−△△CT^ method, △Ct=Ct (target gene)−Ct (endogenous control), △△Ct=△Ct (test group)−△Ct(normal group).^22^ The primer sequences were as follows: *DLGAP1-AS1* forward, 5′-TATGATGATATCAAGAGGGTAGT-3′ and reverse, 5′-TGTATCCAAACTCATTGTCATAC-3′. *DDX59* forward, 5′-GATGTTCCCGTTGATGCTGT-3′ and reverse, 5′-GAGCTTTATTCGAGAGCAAAACT-3′. *GAPDH* forward, 5′-TGGGTGTGAACCATGAGAAG-3′ and reverse, 5′-GTGTCGCTGTTGAAGTCAGA-3′. *ACTB* forward, 5′-GTCAGGTCATCACTATCGGCAAT-3′ and reverse, 5′-AGAGGTCTTTACGGATGTCAACGT-3′. *miR-628-5p* primers, *U6* and *U48* were provided in the TaqMan miRNA reverse transcription kit.

### Cell transfection

Specific short hairpin RNAs (shRNAs) against *DLGAP1-AS1* (sh-*DLGAP1-AS1*#1, sh-*DLGAP1-AS1*#2, and sh-*DLGAP1-AS1*#3), negative control shRNA (sh-NC), pcDNA3.1 vector overexpressing DLGAP1-AS1, and the empty vector were all purchased from GeneChem (Shanghai, China). miR-628-5p mimics, miR-628-5p inhibitor, negative control mimic (NC mimic), and negative control inhibitor (NC inhibitor) were obtained from GenePharma (Shanghai, China). U251, U87MG, or LN229 cells were transfected with Lipofectamine 3000 (Invitrogen, Carlsbad, CA, USA) according to the manufacturer's instructions.

### Cell counting kit-8 (CCK-8) assay

The transfected glioma cells were transferred into a 96-well plate (1 × 10^3^ cells/well), and CCK-8 solution (Dojindo Laboratories, Kumamoto, Japan) was loaded into each well at 0, 24, 48, 72, and 96h, respectively, followed by incubation for 3h. The absorbance of each well was recorded at 450 nm using a microplate reader (BioTek, Winooski, VT, USA).

### 5-Ethynyl-2′-deoxyuridine (EdU) assay

The EdU kit was obtained from RiboBio (Guangzhou, China). The transfected cells were cultured in a 96-well plate (5 × 10^3^ cells/well) for 24h and then incubated with 50 mM EdU reagent for 4h. After discarding the medium, the cells were fixed with 4% paraformaldehyde and incubated for 30 min in the dark with the Apollo fluorescent staining solution. The authors then washed them twice with PBS and incubated with Hoechst staining solution for 20 min. Finally, the authors rinsed the cells three times with PBS and observed and counted them under a fluorescence microscope.

### Transwell assay

Transwell assays were performed using a Transwell system with 8 μm pore size (Corning Incorporated, Corning, NY, USA). In the migration assay, 100 μL of cell suspension (approximately 1 × 10^4^ cells) prepared in serum-free medium was added to the upper chamber, and 500 μL of medium containing 10% FBS was added to the lower chamber. After the cells were cultured for 12h, the cells on the upper side of the membrane were scraped off, and the remaining cells were fixed with 4% paraformaldehyde and then stained with 0.1% crystal violet for 30 min. Subsequently, the number of stained cells was counted under a microscope. For the cell invasion assay, 50 μL of diluted Matrigel (1:8, Sigma-Aldrich, St Louis, MO, USA) was dripped into the upper chamber of the Transwell system to cover the membrane before the inoculation of the cells, and the remaining steps were the same as in the migration assay.

### Dual-luciferase reporter assay

Wild-type and mutant type *DLGAP1-AS1* sequences containing *miR-628-5p* binding sites were synthesized and inserted into the pGL3 vector (Promega, Madison, WI, USA) to construct a wide-type reporter plasmid (DLGAP1-AS1-WT) and mutant reporter plasmid (DLGAP1-AS1-MUT). HEK-293 cells were then co-transfected with the reporter plasmids, miR-628-5p mimic, or control miRNA using Lipofectamine 3000 (Invitrogen, Carlsbad, CA, USA). After 48h, the cells were harvested, and the luciferase activity of each group was measured using a Dual-Luciferase Reporter Assay kit (Promega, Madison, WI, USA).

### Western blot

Total protein was extracted using RIPA buffer (Beyotime, Shanghai, China). Protein samples were quantified using a BCA Protein Assay Kit (Pierce, Rockford, IL, USA), suspended in loading buffer, and denatured. Subsequently, the protein samples were separated via SDS-PAGE and transferred to polyvinylidene fluoride membranes (Life Technologies, Gaithersburg, MD, USA). After blocking with 5% skimmed milk for 1h at room temperature, the PVDF membranes were incubated with primary antibody [Anti-DDX59 (Abcam, ab109592, 1:200) or anti-GAPDH (Abcam, ab8245, 1:2000)] at 4°C overnight and then with secondary antibody (HRP-labeled, Beyotime, 1:2000) for 1.5h at room temperature. Finally, the protein bands were visualized using an ECL Plus kit (Life Technologies, Gaithersburg, MD, USA). GAPDH was used as an endogenous control.

### Statistical analysis

The data are shown as the mean ± standard deviation. The normality of the data was evaluated using the Kolmogorov-Smirnov test. For normally distributed data, the Student's *t*-test or one-way analysis of variance (ANOVA) was employed to analyze the differences between two or multiple groups. For skewed data, comparisons between two groups were performed using the Wilcoxon signed-rank test. In survival analysis, glioblastoma (GBM) patients were divided into two groups: *DLGAP1-AS1* high expression (group cutoff: 50%) and *DLGAP1-AS1* low expression (group cutoff: 50%) (n=81 in each group), and the overall survival rate of GBM patients was analyzed using the Kaplan-Meier method and log-rank test. GraphPad Prism 6.0 was used for drafting, and SPSS software (version 18.0; SPSS, Inc., Chicago, IL, USA) was used for statistical analysis. Statistical significance was set at p < 0.05.

## Results

### DLGAP1-AS1 expression is elevated in glioma tissues and cell lines

**Figure 1.**
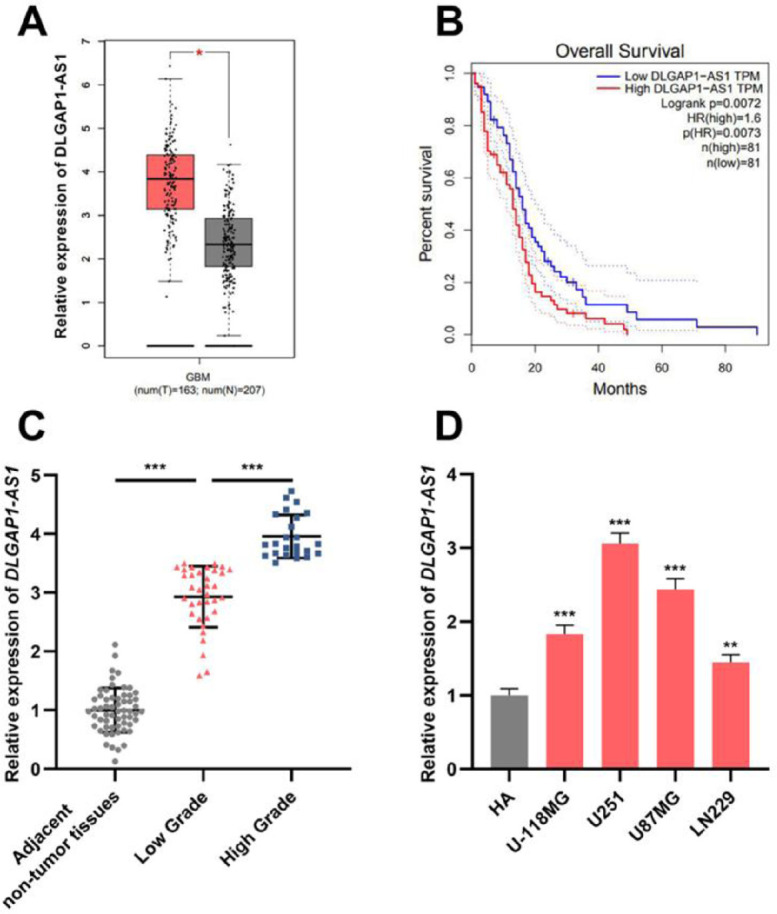
*DLGAP1-AS1* expression is upregulated in glioma tissues and cell lines. A, Bioinformatic analysis was used to analyze *DLGAP1-AS1* expression in GBM and normal brain tissues. B, The GEPIA database was used to perform survival analysis of GBM patients with high and low *DLGAP1-AS1* expression. C, The expression of *DLGAP1-AS1* in glioma tissues and adjacent non-tumor tissues was detected using qRT-PCR (n = 59). D, *DLGAP1-AS1* expression in glioma cell lines (U-118MG, U251, U87MG, and LN229 cells) and normal cell lines (HA cells) was detected using qRT-PCR. The experiments were repeated three times, and the average was recorded. *p < 0.05, **p < 0.01, and ***p < 0.001.

The GEPIA database showed that *DLGAP1-AS1* expression in GBM tissues was higher than that in normal tissues (Fig. 1A). Additionally, Kaplan-Meier survival analysis revealed that high *DLGAP1-AS1* expression was associated with poor survival in GBM patients (Fig. 1B). Next, the authors performed qRT-PCR to determine *DLGAP1-AS1* expression in gliomas and adjacent non-tumor tissues of 59 glioma patients. The results indicated that *DLGAP1-AS1* expression in glioma tissues was remarkably higher than that in adjacent non-tumor tissues, and *DLGAP1-AS1* expression was higher in high-grade tumor samples than in low-grade tumor samples (Fig. 1C and Supplementary Fig. 1A). Consistently, *DLGAP1-AS1* expression was significantly elevated in glioma cell lines (compared to that in HA cells) (Fig. 1D and Supplementary Fig. 1B). In U251 and U87MG cells, *DLGAP1-AS1* was highly expressed, and therefore, U251 and U87MG cells were chosen for the follow-up experiments.

### DLGAP1-AS1 knockdown represses the proliferation, migration, invasion, and EMT of glioma cells

**Figure 2.**
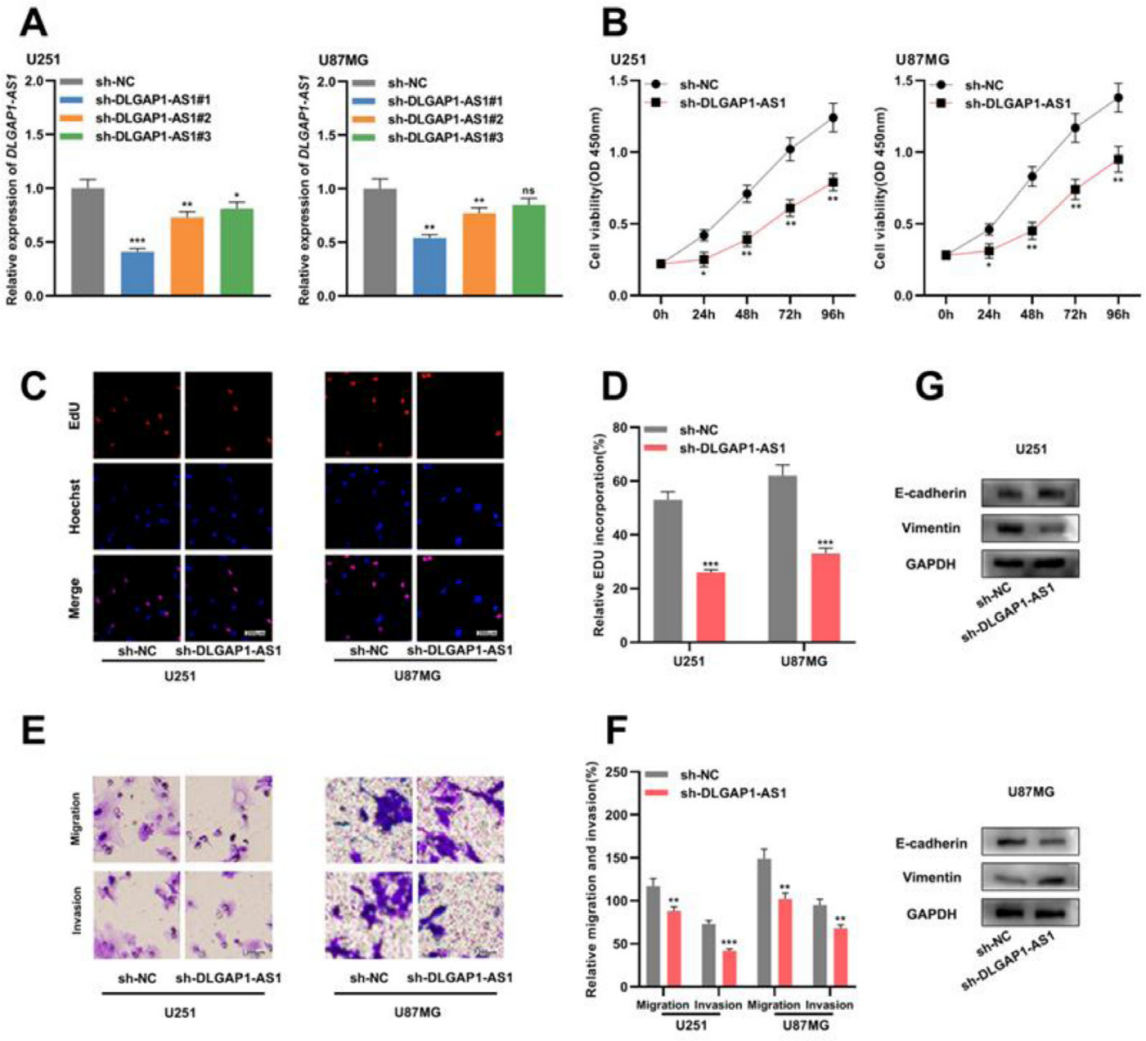
*DLGAP1-AS1* knockdown suppresses the proliferation, migration, invasion, and EMT of glioma cells. A, sh-NC, sh-DLGAP1-AS1#1, sh-DLGAP1-AS1#2, and sh-DLGAP1-AS1#3 were transfected into U251 and U87MG cells to construct low expression models of *DLGAP1-AS1*, and the transfection efficiency was determined using qRT-PCR. (B–D) The proliferation of U251 and U87MG cells transfected with sh-NC or sh-DLGAP1-AS1#1 was detected using CCK-8 (B) and EdU assays (C–D). (E–F) Transwell assay was used to detect the migration and invasion of U251 cells (E) and U87MG cells (F) transfected with sh-NC or sh-DLGAP1-AS1#1. G, The expression levels of EMT-related proteins E-cadherin and vimentin in U251 and U87MG cells transfected with sh-NC or sh-DLGAP1-AS1#1 were detected using western blotting. The experiments were repeated three times, and the average was recorded. *p < 0.05, **p < 0.01, and ***p < 0.001, ns was not statistically significant.

To determine the biological function of *DLGAP1-AS1* in glioma cells, the authors used shRNAs to knockdown *DLGAP1-AS1* expression in U251 and U87MG cells and determined the transfection efficiency using qRT-PCR. It was found that sh-DLGAP1-AS1#1 had the highest efficiency and used it for subsequent experiments (Fig. 2A and Supplementary Fig. 1C). CCK-8 and EdU assays suggested that *DLGAP1-AS1* knockdown significantly reduced the proliferation of U251 and U87MG cells compared to the control group (Fig. 2B-D). The authors performed Transwell assays to evaluate the migration and invasion of glioma cells and found that *DLGAP1-AS1* knockdown markedly reduced the migration and invasion of U251 and U87MG cells (compared with the control group) (Fig. 2E-F). Additionally, western blotting suggested that *DLGAP1-AS1* knockdown increased E-cadherin expression and decreased vimentin expression in glioma cells (Fig. 2G). These findings highlight that *DLGAP1-AS1* knockdown could repress the malignancy of glioma cells.

### DLGAP1-AS1 targets miR-628-5p in glioma

**Figure 3.**
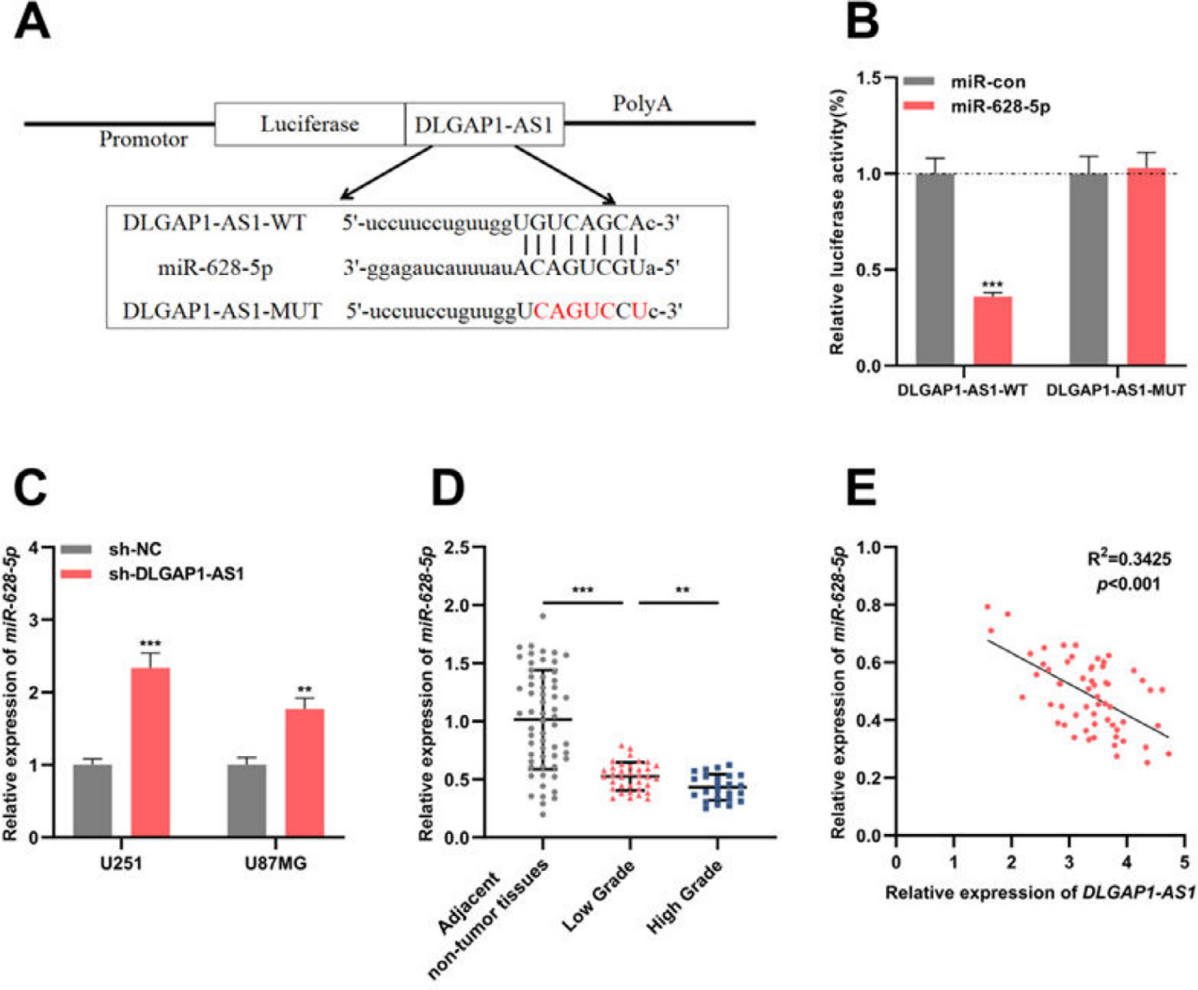
*DLGAP1-AS1* targets *miR-628-5p* in glioma. A, *DLGAP1-AS1*-WT luciferase reporter vector and *DLGAP1-AS1*-MUT luciferase reporter vector were constructed. B, *DLGAP1-AS1*-WT or *DLGAP1-AS1*-MUT luciferase reporter vector and miR-628-5p mimics or control miRNA were co-transfected into HEK-293T cells, and the luciferase activity of the cells in each group was determined. C, qRT-PCR was used to detect the expression of *miR-628-5p* in U251 and U87MG cells transfected with sh-NC or sh-DLGAP1-AS1#1. D, The expression of *miR-628-5p* in glioma cell lines (U-118MG, U251, U87MG, and LN229 cells) and normal cell lines (HA cells) was detected using qRT-PCR. E, Pearson's correlation analysis showed that the expression of *DLGAP1-AS1* and *miR-628-5p* was negatively correlated in glioma tissues. The experiments were repeated three times, and the average was recorded. **p < 0.01 and ***p < 0.001.

To identify the candidate miRNAs that could interact with *DLGAP1-AS1*, the authors searched the StarBase database (version 2.0) and found that the *DLGAP1-AS1* sequence had a binding site for *miR-628-5p* (Fig. 3A). The results of the dual-luciferase reporter assay revealed that *miR-628-5p* restrains the luciferase activity of the DLGAP1-AS1-WT reporter but had no significant effect on the luciferase activity of the DLGAP1-AS1-MUT reporter (Fig. 3B). qRT-PCR showed elevated expression of *miR-628-5p* in U251 and U87MG cells transfected with sh-DLGAP1-AS1, indicating that *DLGAP1-AS1* negatively regulates *miR-628-5p* expression (Fig. 3C and Supplementary Fig. 1D). Additionally, consistent with a previous report,^18^ *miR-628-5p* expression was significantly decreased in glioma tissues (compared to that in adjacent non-tumor tissues) (Fig. 3D and Supplementary Fig. 1E). Next, the authors analyzed the correlation between *DLGAP1-AS1* and *miR-628-5p* expression in glioma tissues using Pearson's correlation analysis, and the authors demonstrated that *DLGAP1-AS1* expression was negatively correlated with *miR-628-5p* expression in glioma tissues (Fig. 3E, R^2^=0.3425), further implying that *DLGAP1-AS1* targets *miR-628-5p* and represses its expression in gliomas.

### Inhibiting miR-628-5p promotes the proliferation, migration, invasion, and EMT of glioma cells

**Figure 4.**
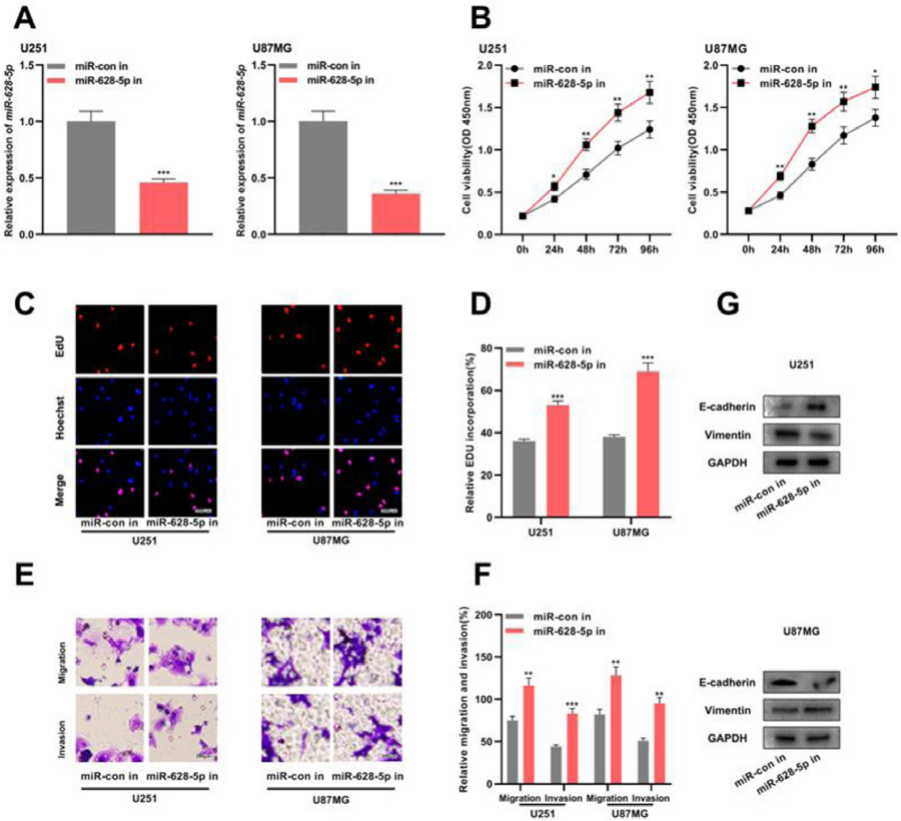
The inhibition of *miR-628-5p* expression promotes the proliferation, migration, invasion, and EMT of glioma cells. A, miRNA inhibitor control (miR-con in) and miR-628-5p inhibitor (miR-628-5p in) were transfected into U251 and U87MG cells to construct models of the inhibition of miR-628-5p expression, and the transfection efficiency was detected using qRT-PCR. (B–D) The proliferation of U251 and U87MG cells was detected using CCK-8 (B) and EdU assays (C-D). (E-F) Transwell assay was used to detect the migration and invasion of U251 (E) and U87MG cells (F). G, Western blot assay was used to detect the expression of EMT-related proteins E-cadherin and vimentin in U251 and U87MG cells. The experiments were repeated three times, and the average was recorded. *p < 0.05, **p < 0.01, and ***p < 0.001.

Previous studies have shown that *miR-628-5p* overexpression inhibits the proliferation of glioma cells.^18^^,^^19^ In the present study, the authors transfected *miR-628-5p* inhibitor into U251 and U87MG cells and verified the transfection efficiency using qRT-PCR (Fig. 4A and Supplementary Fig. 1F). Subsequently, CCK-8, EdU, and Transwell assays and western blotting revealed that the inhibiting *miR-628-5p* promoted the proliferation, migration, invasion, and EMT of U251 and U87MG cells, indicating that *miR-628-5p* exerted tumor-suppressive functions in gliomas (Fig. 4B-G).

### DLGAP1-AS1 regulates glioma cell proliferation, migration, invasion, and EMT via the miR-628-5p/DDX59 axis

**Figure 5.**
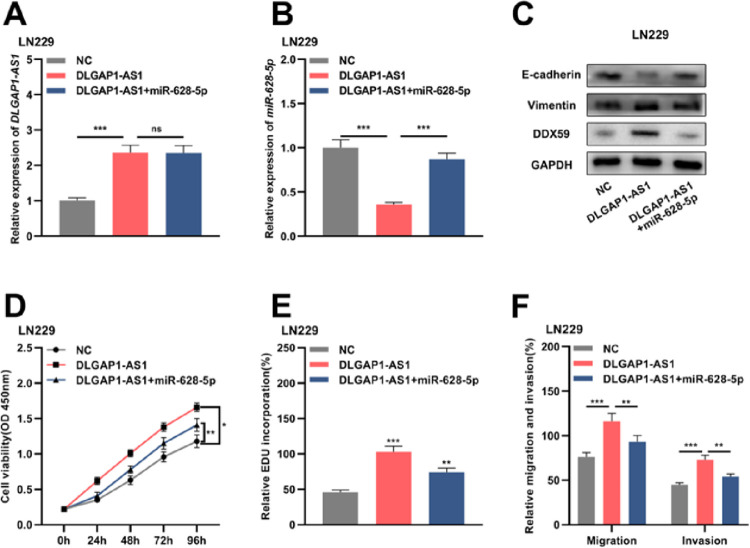
*DLGAP1-AS1* regulates glioma cell proliferation, migration, invasion, and EMT via the miR-628-5p/DDX59 axis. A, The *DLGAP1-AS1* overexpression vector and *DLGAP1-AS1* overexpression vector+miR-628-5p mimic were transfected into LN299 cells, and the expression of *DLGAP1-AS1* was detected using qRT-PCR. B, qRT-PCR was used to detect the expression of *miR-628-5p* in LN299 cells. C, Western blotting was used to detect the expression of *DDX59* and EMT-related proteins E-cadherin and vimentin in LN299 cells. (D–E) The proliferation of LN229 cells was detected using CCK-8 (D) and EdU assays (E). F, Transwell assay was used to detect the migration and invasion of LN229 cells. The experiments were repeated three times, and the average was recorded. *p < 0.05, **p < 0.01, and ***p < 0.001, ns was not statistically significant.

Reportedly, *miR-628-5p* impedes the proliferation of glioma cells by negatively regulating DDX59 expression.^19^ To elaborate on the mechanism of *DLGAP1-AS1* in the biology glioma cells, the authors performed compensation experiments. The authors divided LN229 cells into three groups and transfected them with either the empty vector+NC mimics, pcDNA3.1 overexpressing DLGAP1-AS1+NC mimics, or pcDNA3.1 overexpressing DLGAP1-AS1+miR-628-5p mimics (Fig. 5A-B and Supplementary Fig. 1G). Western blotting was performed to detect DDX59, E-cadherin, and vimentin expression after transfection. The authors found that *DLGAP1-AS1* overexpression promoted the expression of DDX59 and vimentin and repressed the expression of E-cadherin, while miR-628-5p overexpression partially reversed these effects (Fig. 5C). Furthermore, *DLGAP1-AS1* overexpression promoted the proliferation, migration, invasion, and EMT of LN229 cells, and co-transfection with miR-628-5p mimics partially counteracted the functions of *DLGAP1-AS1* (Fig. 5D-F). These experiments indicated that *DLGAP1-AS1* could promote the proliferation, migration, invasion, and EMT of glioma cells by sponging *miR-628-5p* and upregulating DDX59 expression.

## Discussion

LncRNAs play vital roles in human diseases, including tumors.^23^ They are abnormally expressed in diverse tumors, including glioma, and help regulate the malignant biological behaviors of tumor cells, such as proliferation, migration, invasion, apoptosis, and drug resistance.^24^ Many lncRNAs are associated with the pathogenesis and progression of gliomas. For example, increased *LINC00689* expression in glioma tissues and cell lines is associated with rapid deterioration and poor prognosis of patients.^25^ LncRNA *PLAC2* inhibits the nuclear translocation of STAT1, thus reducing *RPL36* expression, inhibiting glioma cell proliferation, and inducing cell cycle arrest.^26^ LncRNA *PVT1* promotes the expression of *BMP2* and *BMP4* by regulating *GREM1* expression and promoting glioma progression.^27^ Herein, the authors confirmed the elevated *DLGAP1-AS1* expression in glioma, which was linked to unfavorable pathological characteristics and poor prognosis of the patients. Functionally, *DLGAP1-AS1* overexpression promoted the proliferation, migration, invasion, and EMT of glioma cells, while its knockdown exerted opposite effects, indicating that *DLGAP1-AS1* is a novel oncogenic lncRNA in glioma and might be a promising therapeutic target.

*miR-628-5p* is a well-known regulator in cancer biology. In most cancers, *miR-628-5p* functions as a tumor suppressor. However, the elevated levels of *miR-628-5p* in osteosarcoma are related to the adverse prognosis of the patients.^28^ The serum of patients with prostate cancer has low levels of circulating *miR-628-5p*, suggesting that *miR-628-5p* is a promising non-invasive biomarker for the diagnosis and prognostic evaluation of prostate cancer. Functionally, *miR-628* reduces the proliferation and invasion of prostate cancer cells by repressing *FGFR2* expression.^29^^,^^30^ In pancreatic ductal adenocarcinoma, *miR-628-5p* suppresses the migration and invasion of cancer cells by repressing Akt/NF-κB signaling.^31^ In gastric cancer, *miR-628-5p* targets *PIN1* to inhibit cancer progression.^32^ In glioma, *miR-628-5p* represses the malignant behavior of glioma cells.^18^^,^^19^ In the present study, the authors found that reduced *miR-628-5p* expression in glioma tissues promoted the proliferation, migration, invasion, and EMT of glioma cells, further confirming the anti-tumor effects of *miR-628-5p* on glioma cells.

*DDX59* is a member of the DEAD/Deah box RNA helicase family.^33^^,^^34^ Reportedly, *DDX59* is highly expressed in lung adenocarcinoma tissues and contributes to the growth of EGFR⁻ lung cancer cells.^35^^,^^36^ *DDX59* knockdown restrained the proliferation of glioma cells, and DDX59 overexpression partially weakened the inhibitory effects of *miR-628-5p*, suggesting that it is also an oncogene in gliomas.^19^ In the present study, the authors proposed a ceRNA network of *DLGAP1-AS1, miR-628-5p,* and *DDX59*. LncRNAs, like ceRNAs, can modulate gene expression by competitively binding to miRNAs, and an imbalance in the ceRNA network can cause diseases.^37^^,^^38^ For example, in gastric cancer, *LINC01133* sponges *miR-106-3p* to upregulate *APC* expression and inhibit cancer progression.^37^ In hepatocellular carcinoma, as a ceRNA, *DLGAP1-AS1* elevates the level of the carcinogenic cytokine IL-6 by sponging *miR-26a/b-5p* and activating the Wnt/β-catenin pathway.^12^ In the present study, through bioinformatics analysis, a dual-luciferase reporter assay, and qRT-PCR, the interaction between *DLGAP1-AS1* and *miR-628-5p* was predicted and validated in glioma cells. The authors also demonstrated that *DLGAP1-AS1* promotes the malignant phenotypes of glioma cells by sponging *miR-628-5p* and elevating *DDX59* expression. These results not only partly explain the mechanism underlying the dysregulation of *miR-628-5p* and *DDX59* in gliomas but also elucidate the mechanism by which *DLGAP1-AS1* participates in glioma progression.

To briefly recapitulate, the present study confirms that *DLGAP1-AS1* is overexpressed in glioma and promotes aggressive cancer progression by regulating the *miR-628-5p*/*DDX59* axis. These findings help clarify the mechanism of glioma progression and provide potential targets for molecular therapy of gliomas. In future studies, animal experiments are needed to verify these results, and it is necessary to enroll more patients from different medical centers to evaluate the potential value of *DLGAP1-AS1* as a biomarker to predict the prognosis of the patients.

## Author contributions

Ke-qi Hu and Xiang-sheng Ao contributed equally to the experimental design and execution, statistical analysis, and manuscript writing.

## Conflicts of interest

The authors declare no conflicts of interest.

## Appendix Supplementary materials

Image, application 1
