# Supplementary material for: Long non-coding RNA DLGAP1 antisense RNA 1 accelerates glioma progression via the microRNA-628-5p/DEAD-box helicase 59 pathway
Source: Clinics (Sao Paulo). 2022 Feb 2;77:100002. doi: 10.1016/j.clinsp.2021.100002 (PMC8903805; doi:10.1016/j.clinsp.2021.100002)

**Clinics_20121-3095 – Supplementary Material**

**APPENDIX**

**Supplementary Figure 1** qRT-PCR was performed to detect the expressions of *DLGAP1-AS1, miR-628-5p*, and *DDX59*. *ACTB* was used as an endogenous control for *DLGAP1-AS1* and *DDX59*, and *U48* was used as the endogenous control for *miR-628-5p*. The experiments were repeated three times, and the average was recorded. *p < 0.05, **p < 0.01, and ***p < 0.001, ns was not statistically significant.


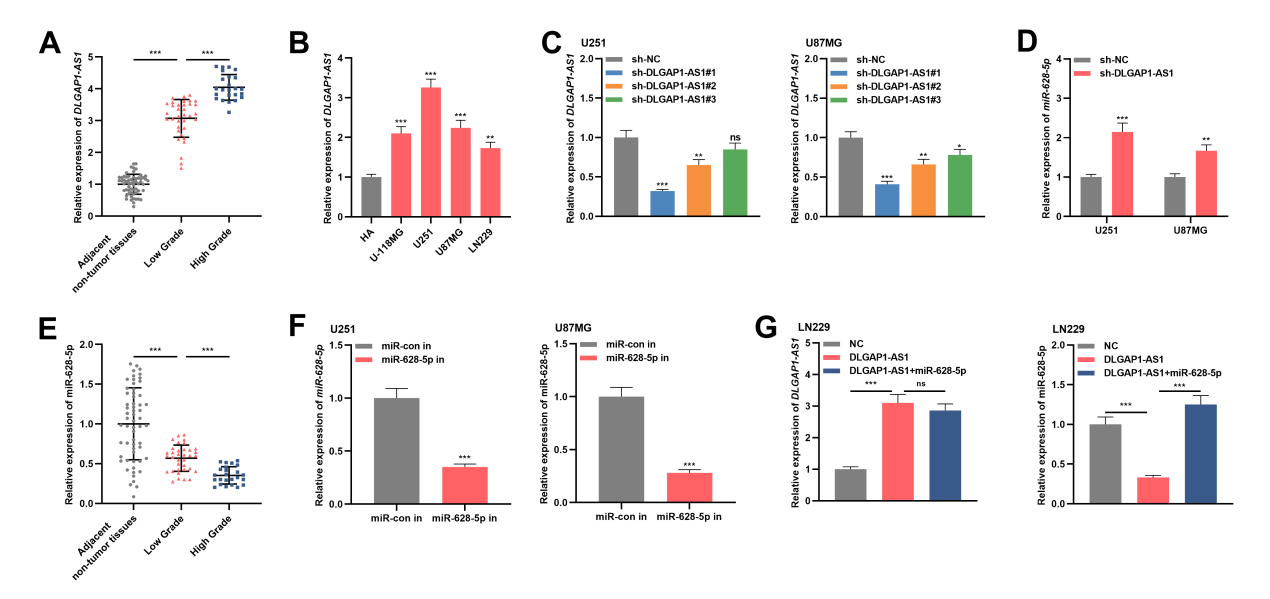

Supplement: Supplementary file 1 [file mmc1.docx]
